# An Update on Imaging in Child Abuse

**DOI:** 10.5334/jbr-btr.1417

**Published:** 2017-11-18

**Authors:** Michael Aertsen

**Affiliations:** 1 University Hospitals Leuven, BE

**Keywords:** Multimodal Imaging, Child abuse, Guidelines

Child abuse is relatively common and recent studies suggest the incidence is rising [1]. It exists in different forms (emotional, neglect, sexual and physical); imaging sometimes plays a pivotal role in physical abuse [2]. Physical child abuse is synonymous to non-accidental injury (NAI) or inflicted injury (II).

Several studies in Europe, the United States and the United Kingdom have demonstrated the great variability in imaging done for NAI and the need for consensus [3,4,5,6]. The most popular guidelines before 2008 were from the American College of Radiologists (ACR) [7]. In 2008 the Royal College of Radiologists, together with the Royal College of Paediatrics and Child Health (RCPCH), published the Standards for Radiological Investigations in Suspected NAI to increase the quality of imaging for this indication [8]. More recently, in 2014, the European Society of Paediatric Radiology has adapted these to be the standard across Europe [9]. The difference between these guidelines and the updated ACR guidelines from 2016, which include the oblique projections of both ribs, are negligible; therefore, this should mean that there is a more homogenous and more evidence based radiological management of these children nowadays [8,9,10,11,12].

The guidelines give recommendations about the classical skeletal survey at admission as well as the necessity of follow-up imaging or the use of bone scintigraphy, computer tomography (CT) or magnetic resonance imaging (MRI) as additional imaging modalities [8].

The skeletal survey should consist of at least 21 x-rays imaging the entire skeleton in separate projections with appropriate incidences and technical settings, often requiring two radiographers (Table 1). This skeletal survey should be taken completely in every suspected NAI in a child less than two-years-old, when the child is greater than two-years-old other elements should be considered (history, clinical examination, index of suspicion, etc.). The *basic* skeletal survey can and should be extended with additional lateral and coned views of the joints or suspicious bones as they can depict/confirm subtle abnormalities more easily. Follow-up imaging should be done at 10–14 days after the initial survey. [8,11,12] The region that should be imaged at follow-up is still subject of debate in the current literature. Follow-up skeletal survey may demonstrate a periosteal reaction confirming the initial suspected fracture or highlighting other previously unsuspicious sites. Important to know is that CMLs can heal with or without new bone formation depending on the presence of periosteal stripping at the time of trauma [11].

**Table 1:** Standard Skeletal Survey for Suspected Child Abuse.

**Skull**	

	AP and Lateral view
	*Even when head CT is being performed [8,10,11,12] although debatable since more recent studies [10]*
	*Townes view when indicated clinically*
**Chest**	

	AP *including the clavicles*
	Left and right oblique views of the ribs*
**Limbs**	

	AP of both upper arms, both forearms, both femurs and both lower legs
	PA of both hands
	DP of both feet
**Spine**	

	Lateral *with separate projections if necessary for cervical, thoracic and thoracolumbar regions*
	*AP views of the part which is not seen on the AP view of the abdomen and chest*
	*AP view of the cervical spine only after discussion with the radiologist*
**Abdomen**	

	AP view *including pelvis and both hips*

*This was a major difference between the former ACR guidelines and the RCR Guidelines but has now been included in the Revised ACR Guidelines 2016.

Fractures that are known to be related to inflicted injury are classic metaphyseal lesions (CML), posterior rib fractures, complex skull fractures and spiral or oblique humeral fractures in children less than 15 months of age. Other suspicious fractures in unusual locations are fractures in the scapula, sternum or spinous process because they require significant force and cannot be fractured by normal handling. Fractures of the ischiopubic rami have been associated with sexual abuse; often these are associated with multiple injuries [11,12].

Dating of fractures can be important in cases of NAI but is an inexact science with only sparse evidence in the literature. The estimates of time are done in weeks rather than days and there is significant overlap in the features of bone healing. When multiple fractures are present, however, a radiologist should be able to differentiate new from old fractures [13].

There are situations in which a bone scan is the additional modality of choice as it can become positive within seven hours after bony injury [14]. This is usually the case when follow-up imaging is not an option, either because of safety concerns for the child or failure to attend repeat examinations is felt likely. Scintigraphy is, however, complementary to radiography because it may highlight unsuspicious sites on radiography but may be of less value for metaphyseal and skull fractures. Suspicious sites on the scintigraphy will always need to be confirmed on x-ray [8,14].

Every child less than one-year-old with evidence of physical abuse should have neuro-imaging with MRI as should every child (of any age) with evidence of physical abuse and encephalopathic or focal neurological signs or haemorrhagic retinopathy [8].

CT is mainly used in the acute setting of neurological injury, once the child is stabilized, due to its availability and its high sensitivity for acute intracranial haemorrhage as well as secondary parenchymal abnormalities. If abnormal or persisting clinical neurological signs are present with equivocal CT, MRI should be performed early (e.g. day 3–5) [8,10,13]. Late sequelae should be investigated by MRI at 3 to 6 months [8,13].

A paediatric radiologist or a general radiologist with special interest in paediatric radiology should repot the examination [8]. This might not reflect the clinical reality and thus the guidelines mention the value of local and regional networks allowing for easy access to second opinions in equivocal cases. Double reporting is not mentioned explicitly in the guidelines but is said to be vital. It reduces the risk of missed injuries, allows learning from one another and spreads the burden of the impact of these examinations on several radiologists [11,12]. The report should be written and contain a level of confidence as well as a summary of the justification of for the opinion [8]. In addition to the written report a verbal report should be given to the referring physician. Ideally, there should be a joint revision of the images by the radiologist and the clinical team [8,11,12].

Sometimes the radiologist is the first to suspect NAI and in these circumstances action must be undertaken to ensure the child’s safety by contacting the referring clinician and his team [8].
